# Intact Transition Epitope Mapping—Serological Inspection by Epitope EXtraction (ITEM—SIX) †

**DOI:** 10.3390/molecules28073092

**Published:** 2023-03-30

**Authors:** Agatino Zammataro, Cornelia Koy, Manuela Ruß, Claudia Röwer, Michael O. Glocker

**Affiliations:** 1 Proteome Center Rostock, University Medicine Rostock and University of Rostock, Schillingallee 69, 18059 Rostock, Germany; 2 Department of Chemical Sciences, University of Catania, Viale Andrea Doria 6, 95125 Catania, Italy

**Keywords:** blood serum, surrogate seroconversion, immune complex analysis, epitope mapping, nanoESI mass spectrometry

## Abstract

Precision medicine requests accurate serological inspections to precisely stratify patients for targeted treatment. Intact transition epitope mapping analysis proved surrogate seroconversion of a model organism’s serum when spiked with a monoclonal murine anti-Ovalbumin antibody (mAb) with epitope resolution. Isolation of the IgG fraction from blood serum applied two consecutive protein precipitation steps followed by ultrafiltration and resulted in an ESI-MS analysis-ready IgG preparation. For epitope mapping by epitope extraction, the Ovalbumin antigen was digested with trypsin. After desalting, the peptide mixture was added to the ESI-MS-ready IgG preparation from mAb-spiked serum and the solution was incubated to form an immune complex between the Ovalbumin-derived epitope peptide and the anti-Ovalbumin mAb. Then, the entire mixture of proteins and peptides was directly electrosprayed. Sorting of ions in the mass spectrometer’s gas phase, dissociation of the immune complex ions by collision-induced dissociation, and recording of the epitope peptide ion that had been released from the immune complex proved the presence of the anti-Ovalbumin mAb in serum. Mass determination of the complex-released epitope peptide ion with isotope resolution is highly accurate, guaranteeing high specificity of this novel analysis approach, which is termed Intact Transition Epitope Mapping—Serological Inspections by Epitope EXtraction (ITEM—SIX).

## 1. Introduction

Serology is classically defined as the study of proteins, predominantly antibodies, in blood and other body fluids [1]. Serology has been used to provide information about an individual’s exposure and immunity to specific pathogens, e.g., to test for past and current viral infections [2] or to complement case-based surveillance for determining disease burden in order to gain an overview on epidemiology of infectious diseases [3]. Despite not being quantitative [4], current serology is increasingly viewed as a powerful source of molecular information that could complement classical case-based disease surveillance and help guide public health policy [3]. As the antibody repertoire in body fluids is shaped by the record of exposure to exogenous factors as well as by endogenous host-intrinsic factors, such as self-antigens [1], antibody tests from donor blood are used to estimate an individual’s immune reactiveness, e.g., antigen recognition via indirect pathway by recipient antigen presenting cells, autoreactive T cell activation, autoreactive B cell activation, T helper 17 cell differentiation, loss of self-tolerance, and epitope spreading phenomena [5]. Medically important serological determinations comprise estimation of the proportion of women with and without tubal factor infertility who have had previous *Chlamydia trachomatis* infection, thereby assessing the associated risk of infertility [6]. In addition, the phenomenon of antibody-mediated rejection of transplants requests serological investigations [7], and an attempt to estimate donor transplant complications has been undertaken by determining and correlating autoantibody titers of patients with negative post-transplant outcome [8]. Both alloimmunity and autoimmunity have been found to play synergistic roles in the formation of non-HLA antibodies, and non-HLA mismatches between donors and recipients provided valuable information regarding the role of genetics in non-HLA antibody immunity and development [5]. Moreover, testing allergen antibody profiles in children has been suggested as a superior diagnostic readout as compared to focused single-allergen-specific antibody analysis [9]. Allergy blood tests have become indispensable [10], and multiplexing capabilities of serological test systems are of particular interest [11].

Mass spectrometry provides multiplexing capabilities [12] and has been used to characterize autoantibodies in autoimmune diseases, exemplified by systemic lupus erythematosus (SLE) and Sjögren’s syndrome (SS), by identifying the immunoglobulin variable region subfamily usage as well as by characterizing mutational profiles at the molecular level [13]. For expression profiling of human autoantibodies, a quantitative MRM-MS platform had been installed for targeted identification and monitoring of expression of pathogenic clonotypes in patient sera over time [14]. Mass spectrometric antibody sequencing has been applied to identify the presence of therapeutic and endogenous antibodies in donor blood [15]. Moreover, IgG antibodies were isolated from serum and the donors’ light chain clonalities were determined by mass spectrometric sequencing using CID fragmentation, thereby creating individual compositional profiles [16].

Significant advances in mass spectrometric epitope mapping protocols have been reached with the two most commonly used methods: epitope extraction and epitope excision [17,18,19]. These epitope mapping methods have reached advanced stages either through automation of solution handling procedures [20] or via reducing in-solution handling to mixing of antigens and antibodies in compatible electrospray ionization (ESI) mass spectrometry buffer solutions [21,22]. For the latter, direct transition of immune complexes from condensed phase into the gas phase, which is routinely realized by ESI-MS [23,24], i.e., desorption and simultaneous ionization, has been recognized as the key process for successful mass spectrometric epitope mapping. Mass spectrometers allow separation of ions based on their *m*/*z* values and this has found application for simple, robust, fast, and easy-to-apply antibody–antigen binding studies, termed intact transition epitope mapping (ITEM; [22]). ITEM—ONE identifies epitopes by determining the mass of the extracted epitope peptide upon dissociation of immune complex ions in the gas phase [22,25]. Determining kinetic and thermodynamic properties, i.e., quantitative traits of immune complexes, is possible using the newly developed ITEM—TWO method [26,27]. Recently, ITEM—THREE had been developed by us to identify unknown antigenic determinants on an antigen surface by sequencing complex-released epitope peptides followed by database search [28]. Above all, the most recently developed ITEM—FOUR procedure targets epitope fine mapping, i.e., the determination of recognition motifs within an epitope peptide with amino acid residue resolution [29,30].

The objective of this work is focused on seroconversion analysis using mass spectrometry, more precisely, intact transition epitope mapping (ITEM), as a readout. As a study object, we chose rabbit serum into which had been spiked a monoclonal murine anti-Ovalbumin antibody (mAb). Within this proof-of-principle study, the IgG fraction had been isolated from the mAb-spiked serum following a newly developed antibody preparation protocol without applying affinity chromatography. Then, the ESI-MS-ready IgG preparation (solution 1) was mixed with a tryptic Ovalbumin peptide mixture, which contained the epitope peptide (solution 2). For identification of the epitope, the ITEM—ONE procedure was applied. The in-solution-formed immune complex, together with all other components of the mixture (solution 3), was subjected to electrospray ionization. Presence of the anti-Ovalbumin antibody in the mAb-spiked serum was proven by recording the extracted and (in the gas phase from the immune-complex) released epitope peptide ion. This novel seroconversion analysis approach is termed Intact Transition Epitope Mapping—Serological Inspections by Epitope Extraction (ITEM—SIX).

## 2. Results

### 2.1. IgG Preparation from Blood Serum

**Figure 1:**
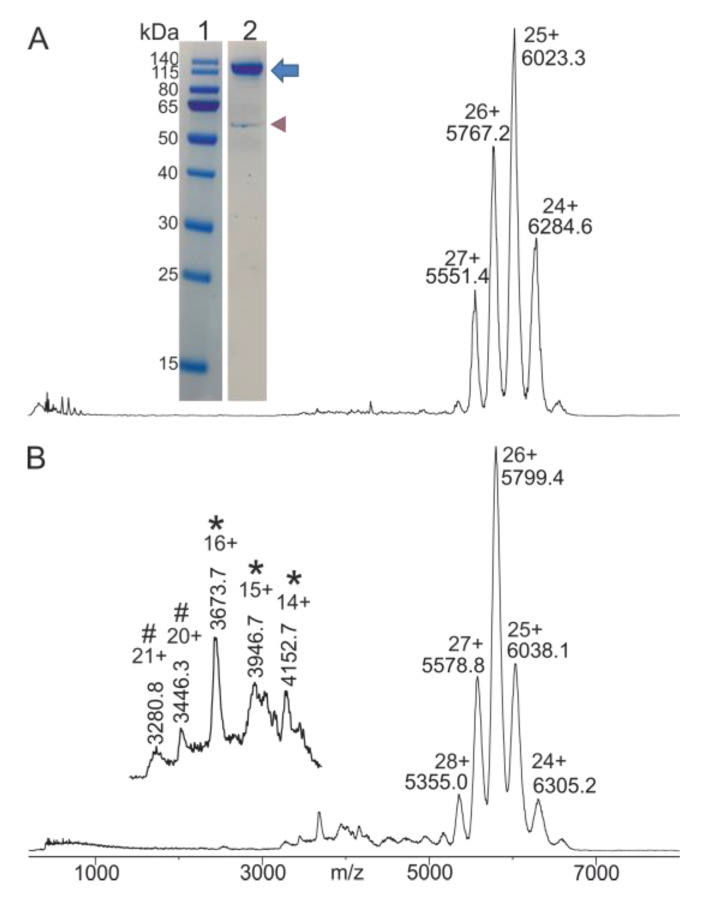
Characterization of an IgG mix prepared from blood serum. (**A**) Nano-ESI mass spectrum of IgG mix (solution 0). Charge states and *m*/*z* values are provided for the multiply charged IgG ion signals. Quadrupole vacuum: 3.9 × 10^−5^ mbar. Insert: SDS-PAGE analysis of IgG mix prepared from serum under non-reducing conditions. Lane 1: molecular mass marker (PageRuler Prestained Protein Ladder). Apparent molecular masses are shown at the left. Lane 2: from blood-serum-isolated IgG mix (1 µg protein). Proteins were stained with colloidal Coomassie Brilliant Blue. The blue arrow points to the IgG band. The purple arrow head indicates residual serum albumin. (**B**) Nano-ESI mass spectrum of IgG mix (solution 0). Charge states and *m*/*z* values are provided for the multiply charged IgG ion signals. Quadrupole vacuum: 3.2 × 10^−5^ mbar. Insert: ion signals (*m*/*z* values and charge states) of residual monomeric serum albumin (#) and tetrameric transthyretin (*). Protein concentration is 0.34 µg/µL. 2.5 µL were loaded. Solvent: 200 mM ammonium acetate, pH 6.7. Recording time per spectrum is 1 min.

IgG was prepared from blood serum using a three-step protocol (see Section 4), which allowed to enrich the immunoglobulin content in serum while depleting other serum proteins by two subsequent partial precipitations. Upon re-solubilization of the immunoglobulins from the second precipitate, the buffer was exchanged by ultrafiltration, thereby providing a nearly pure IgG preparation dissolved in ammonium acetate buffer with a pH of 6.7. Since ammonium-acetate-buffered protein-containing solutions are compatible with electrospray ionization, the IgG preparation (solution 0) was directly subjected to nanoESI-MS analysis without any further purification (Figure 1).

The mass spectrum of the IgG preparation (solution 0) showed the typical ion signal distribution of IgG, and, from the multiply charged ion signals, the 25+ ion signal was the one with the highest intensity. An average molecular mass of 150,080 (±957) was experimentally determined (Table S1), which stands in agreement with known molecular masses of mammalian IgG. The broad ion signals indicate a heterogenous composition of an unknown number of IgG in the mixture. Analysis of the IgG preparation (solution 0) by SDS-PAGE under non-reducing conditions provided a sharp and strongly stained band (Coomassie staining) at an apparent molecular mass of 150 kDa together with a faint band at apparent molecular mass of ca. 60 kDa (Figure 1A, insert). The presence of traces of other serum proteins in the IgG preparation (solution 0) was also found by nanoESI-MS analysis upon slightly altering measurement conditions. With a steeper pressure gradient between the ESI source (atmosphere: ambient pressure) and the first mass analyzer (quadrupole vacuum: 3.2 × 10^−5^ mbar), in-source desolvation processes were enhanced and afforded multiply charged ion signals with low intensities of two serum proteins, i.e., tetrameric transthyretin and monomeric serum albumin (Figure 1B), as well as a shift in the charge state envelope of the IgG ions [25]. In fact, presences of these two serum proteins as well as of haptogloblin, serotransferin, and apolipoprotein A1 [31,32], all in low abundances, were detected upon silver staining of the proteins after SDS-PAGE analysis (Figure S1). Despite the presence of a few serum proteins in low abundances, the IgG preparation (solution 0) that had been obtained by the here applied fractionated precipitation and rebuffering procedure was considered pure enough to perform mass spectrometric seroconversion investigations.

### 2.2. Mass Spectrometric Characterization of Starting Materials

**Figure 2:**
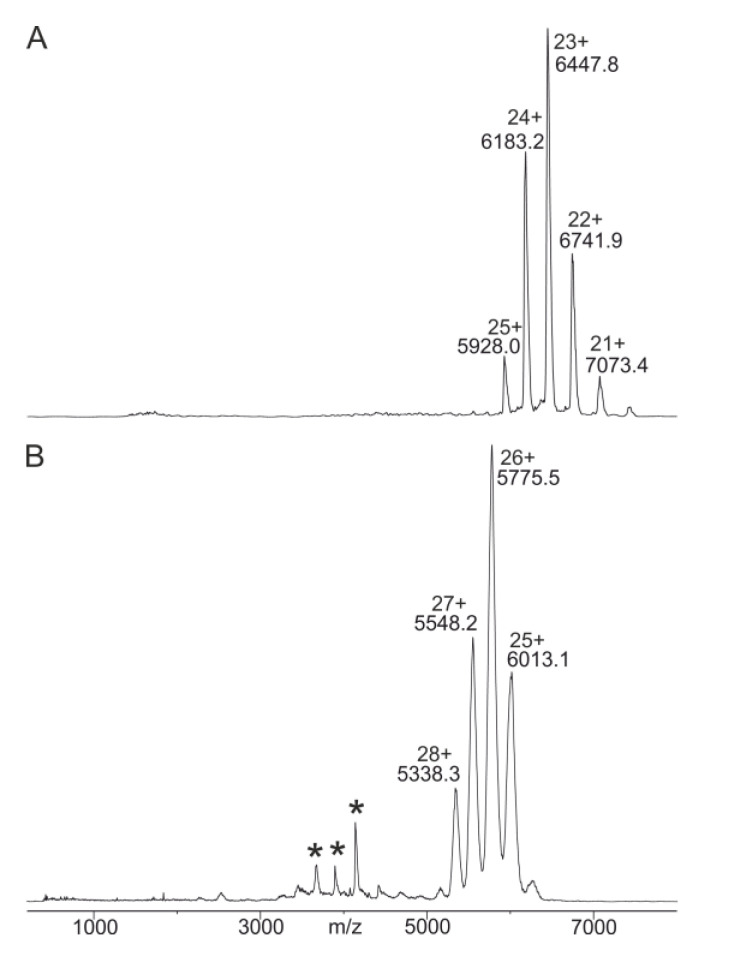
Characterization of anti-Ovalbumin antibody and IgG mix prepared from converted serum. (**A**) Nano-ESI mass spectrum of anti-Ovalbumin antibody (solution 1a). Charge states and *m*/*z* values are provided for the multiply charged IgG ion signals. Protein concentration is 0.28 µg/µL. 2.5 µL were loaded. (**B**) Nano-ESI mass spectrum of IgG mix (solution 1b) prepared from mAb-spiked serum. Charge states and *m*/*z* values are provided for the multiply charged IgG ion signals. Ion signals of residual tetrameric transthyretin (*) are marked. Protein concentration is 0.17 µg/µL. 2.5 µL were loaded. Solvent: 200 mM ammonium acetate, pH 6.7. Quadrupole vacuum: 3.2 × 10^−5^ mbar. Recording time per spectrum is 1 min.

As a test system for the proof-of-concept study, we selected the murine anti-Ovalbumin antibody [6C8], a commercially available monoclonal antibody (mAb) that recognizes chicken Ovalbumin (P01012), which itself is a readily available food allergen. Mass spectrometric characterization of the anti-Ovalbumin mAb dissolved in ammonium acetate buffer (solution 1a) by nanoESI-MS afforded a mass spectrum with the typical charge distribution of antibody ion signals with the most abundant 23+ charged ion signal (Figure 2). From the multiply charged ion signals, a molecular mass of 148,329 (±128) was experimentally determined (Table S1).

For surrogate seroconversion, the anti-Ovalbumin antibody had been spiked into rabbit serum. Then, the mAb-spiked serum was subjected to IgG preparation using the newly developed fractionated precipitation procedure and the respective IgG mixture (solution 1b) was analyzed by nanoESI-MS (Figure 2). The mass spectrum of the IgG mixture from mAb-spiked serum (solution 1b) nearly completely resembled the mass spectrum of the IgG mixture from serum (solution 0) with the 26+ charged antibody ion signal as the most intense ion signal in the mass spectrum. Again, ion signals from tetrameric transthyretin had been detected in the mixture with low intensities. The IgG preparation from mAb-spiked serum was considered suitable for performing mass spectrometric seroconversion investigations.

**Figure 3:**
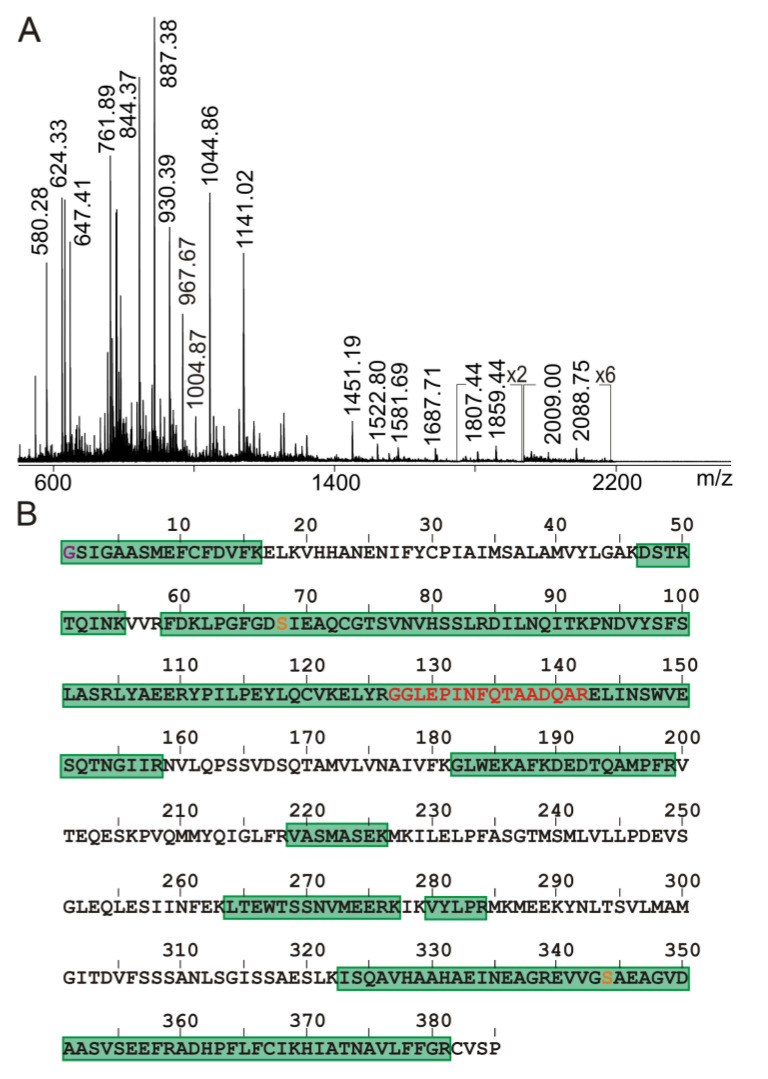
Characterization of the Ovalbumin antigen. (**A**) NanoESI mass spectrum of ions from Ovalbumin upon tryptic digestion (solution 2′). Selected ion signals are labeled. Ion intensity multiplication factors are provided. Peptide concentration is 0.28 µg/µL. 2.5 µL were loaded. Solvent: 200 mM ammonium acetate, pH 6.7. Recording time is 1 min. (**B**) Amino acid sequence of chicken Ovalbumin (P01012; single letter code). Sequence parts that match with peptide ion signals are shaded in green (sequence coverage: 60%). Modified amino acid residues are highlighted in orange (cf. Table S2). The epitope peptide sequence is printed with red letters.

Next, the Ovalbumin antigen solution (solution 2) was inspected for its molecular integrity, and SDS-PAGE analysis under non-reducing conditions provided a single strongly stained protein band at an apparent molecular mass of ca. 45 kDa at the expected location in the gel (Figure S2). Upon tryptic digestion of the reduced and alkylated Ovalbumin and by mass spectrometric peptide mapping of the obtained peptide mixture (solution 2′), a sequence coverage of 60% was determined (Figure 3 and Table S2).

Peptide ion signals with high intensities were recorded in the mass range below *m*/*z* 1400. However, despite the fact that peptide ion signals above *m*/*z* 1400 were present in only low intensities, they were unambiguously assigned to the respective partial sequences of chicken Ovalbumin. The known post-translational modifications, such as acetylation of the N-terminal glycine residue (G1) and phosphorylations at amino acid residues S68 and S344, were confirmed. Despite the fact that (i) there remained unassigned peptide ion signals (Table S3) and (ii) the sequence coverage of assigned peptide ion signals was not 100%, the obtained peptide mix (solution 2′) was considered suitable for both epitope mapping by ITEM—ONE and mass spectrometric seroconversion investigations.

### 2.3. Anti-Ovalbumin Antibody Epitope Mapping by ITEM—ONE Analysis

An epitope mapping experiment typically starts with testing immune complex formation efficiency with a standard immune analysis assay, such as Western blotting. The after SDS-PAGE separation blotted Ovalbumin (solution 2) was readily decorated by the anti-Ovalbumin mAb (solution 1a) and detected upon addition of the fluorescence-labeled secondary anti-murine IgG antibody using a fluorescence imaging system (Figure S3).

Likewise, immune complex formation was confirmed by nanoESI-MS analysis after mixing the anti-Ovalbumin mAb (solution 1a) with the Ovalbumin-derived peptide mixture (solution 2′) upon electrospraying the entire mixture. The mass spectrum was recorded under ITEM—ONE conditions, which means that transmission of ions with low *m*/*z* values (cut-off setting: x = *m*/*z* 2000) was suppressed and only ions with high *m*/*z* values (typically *m*/*z* values > 1600 (>0.8x)) were able to reach the detector with more or less unmuted intensities. The ITEM—ONE mass spectrum showed multiply charged ion signals (21+ to 25+) of the antibody, which were accompanied by multiply charged satellite ion signals (21+ to 25+) on the high mass sides of the ion signals (Figure S4). From the latter ion signals, an immune complex mass of 149,803 (±63) Da was experimentally determined (Table S1), indicating the addition of one epitope peptide to one antibody molecule. Since accuracy of the mass determination of such large molecules and/or immune complexes is not precise enough to unambiguously identify the bound epitope peptide, additional experiments are needed for unequivocally assigning the anti-Ovalbumin antibody’s epitope.

**Figure 4:**
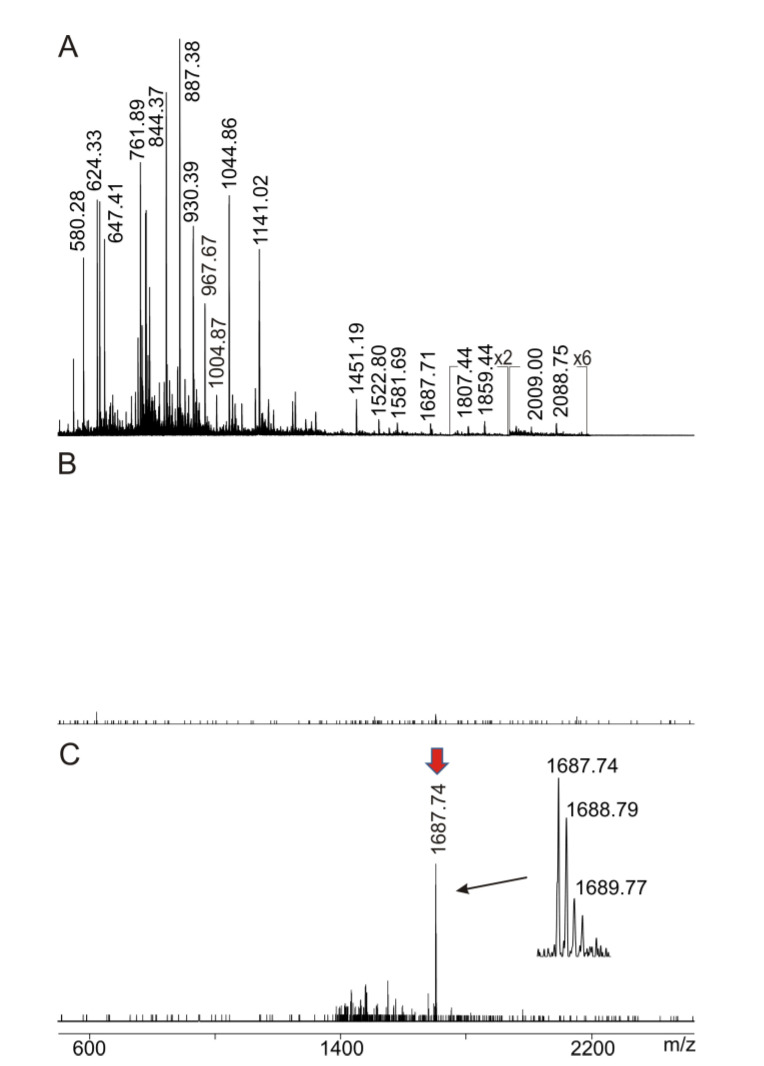
ITEM—ONE analysis. (**A**) NanoESI mass spectrum of ions from Ovalbumin upon tryptic digestion (solution 2′). Ion intensity multiplication factors are provided. (**B**,**C**) NanoESI mass spectrum of ions from mixture (solution 3a) of tryptically digested Ovalbumin (solution 2′) with anti-Ovalbumin mAb (solution 1a). Ion transmission below *m*/*z* 1450 is completely suppressed. (**B**) ∆CV: 3 V. (**C**): ∆CV: 20 V. Selected *m*/*z* values are provided. The red arrow points to the complex-released epitope peptide ion signal. The insert in (**C**) shows the isotope pattern of the complex-released peptide ion signal. Protein concentration is 0.22 µg/µL. 2.5 µL were loaded. Solvent: 200 mM ammonium acetate, pH 6.7. 1 min recording time per spectrum. 30% baseline subtraction.

Therefore, ITEM—ONE analysis was applied. Immune complex dissociation in the collision cell of the mass spectrometer and subsequent mass determination of the complex-released peptide ion provided the required information with the requested accuracy. After increasing the collision cell voltage difference (ΔCV), the complex-released epitope peptide was analyzed by the mass spectrometer’s second mass analyzer with isotope resolution (Figure 4).

Since the singly protonated ion signal with *m*/*z* 1687.74 was the only ion signal that appeared with strongly increased intensity in the low mass range of the mass spectrum upon increasing ΔCV, this ion signal was determined to represent the epitope peptide that had been released from the immune complex by CID. The mass of the singly protonated ion signal with *m*/*z* 1687.74 matched precisely with the calculated mass of the chicken Ovalbumin partial amino acid sequence GGLEPINFQTAADQAR (sequence range 127–142). This peptide had been produced by tryptic cleavage at amino acid residues R126 and R142 (cf. Figure 3 and Table S2).

As a negative control experiment, the Ovalbumin peptide mixture (solution 2′) was mixed with the IgG preparation that had been prepared from blood serum (solution 0). Under ITEM—ONE conditions, i.e., with blocking transmission of low *m*/*z* ions and increasing ΔCV in the collision cell, no ion signals appeared in the low mass range, whereas the ion signals with high *m*/*z* values, which belonged to the antibodies, were readily detected (Figures S5 and S6). Obviously, antibodies from rabbit blood serum were not able to bind to any of the Ovalbumin-derived peptides and, consequently, no immune complexes had formed in solution, which is consistent with Western blot data (Figure S3).

To confirm assignment of the epitope peptide ion signal at *m*/*z* 1687.71 to the chicken Ovalbumin partial amino acid sequence GGLEPINFQTAADQAR, a mass spectrometric fragmentation experiment of this ion was performed. The MS/MS spectrum showed intense Y-type ion signals that were unambiguously assigned by comparing the experimentally determined masses with the fragments’ theoretical masses (Figure S7), confirming the partial amino acid sequence assignment of the precursor peptide.

### 2.4. Intact Transition Epitope Mapping—Serology Inspection by Epitope EXtraction (ITEM—SIX)

**Figure 5:**
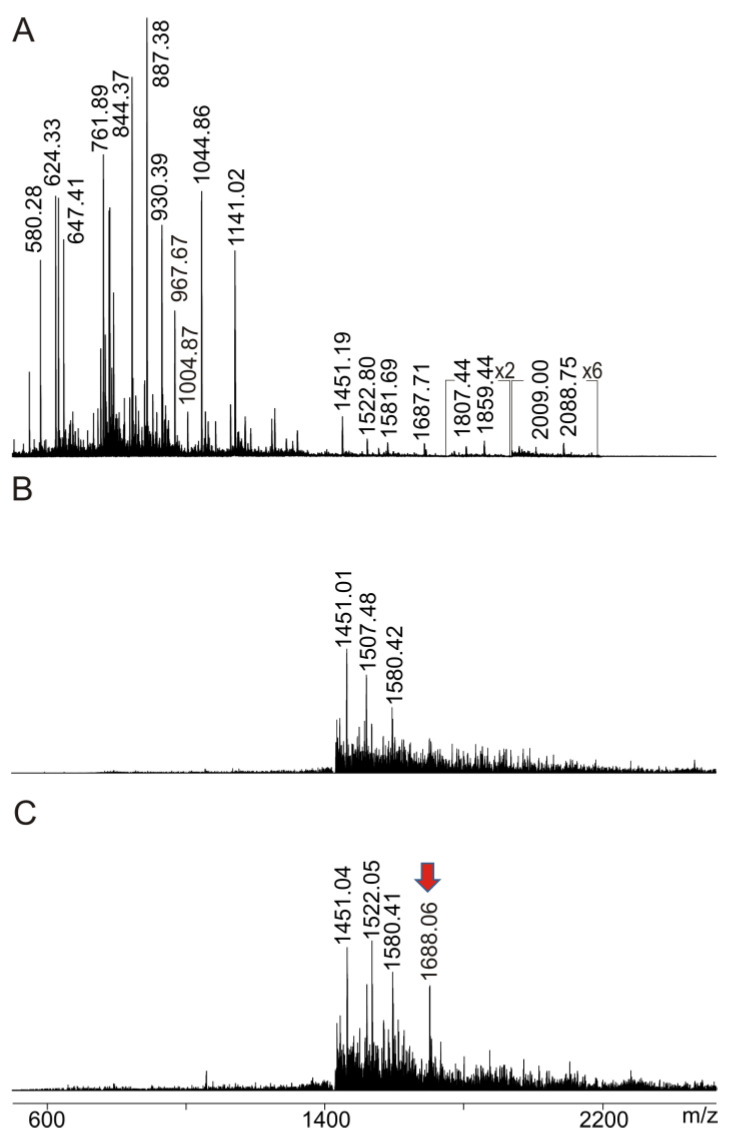
ITEM—SIX analysis. (**A**) NanoESI mass spectrum of ions from Ovalbumin upon tryptic digestion (solution 2′). Ion intensity multiplication factors are provided. (**B**) NanoESI mass spectrum of ions from mixture (solution 3b) of tryptically digested Ovalbumin (solution 2′) with IgG mixture prepared from mAb-spiked blood serum (solution 1b). Ion transmission below *m*/*z* 1450 is completely suppressed. (**B**) ∆CV: 3 V. (**C**) ∆CV: 20 V. Selected *m*/*z* values are provided. The red arrow points to the complex-released epitope peptide. Protein concentration is 0.81 µg/µL. 2.5 µL were loaded. Solvent: 200 mM ammonium acetate, pH 6.7. Recording time per spectrum is 1 min. 50% baseline subtraction.

Encouraged by both, the very clear ITEM—ONE epitope mapping result and the newly developed IgG preparation procedure from blood serum, we moved forward and started a mass spectrometric seroconversion investigation. For surrogate seroconversion, the anti-Ovalbumin antibody was spiked into blood serum and the mAb-spiked serum was subjected to IgG preparation. The Ovalbumin-derived peptide mixture (solution 2′) was mixed with the IgG preparation obtained from mAb-spiked blood serum (solution 1b) and the entire mixture (solution 3b) was analyzed by nanoESI-MS using ITEM conditions (Figure 5).

The mass spectrum that was recorded at low ΔCV showed in the low *m*/*z* range (*m*/*z* 500–2500) just a few ion signals above *m*/*z* 1400, all with very little intensities, indicating that blocking of ions with *m*/*z* values below *m*/*z* 2000 was incomplete for ions with *m*/*z* values higher than 1400. By contrast, transmission of ions with *m*/*z* values below 1400 was completely suppressed. Nevertheless, upon increasing ΔCV, there appeared an additional singly protonated ion signal at *m*/*z* 1688.06, which is the only ion signal that increased in intensity in the mass spectrum and thus is assigned as a complex-released peptide ion signal. No other low abundant ion signal changed intensity with increasing ΔCV.

As with all other experiments in which antibodies were analyzed in the presence of peptide mixtures, their multiply charged ion signals were recorded with *m*/*z* values above *m*/*z* 5000 (Figure S8). Because of intrinsic heterogeneity the multiply charged ion signals of the IgG mix alone are rather broad and, hence, no immune complex ion signals could be distinguished.

The negative control experiment was repeated by mixing the Ovalbumin peptide mixture (solution 2′) with the IgG preparation which had been prepared from blood serum (solution 0). Again, an increase of ΔCV in the collision cell caused no additional ion signals to appear in the low mass range. The pattern of incompletely suppressed low abundant ion signals maintained the same. Multiply charged antibody ion signals with high *m*/*z* values which traversed to the instrument’s detector in unmuted fashion were recorded as expected (Figures S9 and S10). 

## 3. Discussion

Ovalbumin from chicken is a major allergen against which susceptible humans may generate antibodies (IgG and/or IgE), leading to food intolerance and/or allergic reactions towards the chickens’ eggs’ ingredients [33,34]. The epitope peptide GGLEPINFQTAADQAR identified by us (sequence range 127–142) of the commercially available anti-Ovalbumin antibody (C68) contains the amino acid sequence LEPINF, which is the epitope of the hybridoma clone “HYB 94-06” [35], suggesting a relation—or even identity—of the commercially available antibody to the respective hybridoma clone from the Danish lab. Interestingly, the Ovalbumin-derived peptic peptide YRGGLEPINF was found to induce vasodilatory and anti-hypersensitive effects when orally administered to rats [36]. 

Because of targeting an antigen’s epitope, ITEM—SIX has the potential to move current antigen-based serology to the next level of future epitope-based serological investigations. This move towards fine-tuning of biomedical assays, which detect an antibody via its defined recognition motif in a crude mixture, such as blood serum, enables to dissect patient cohorts not only according to the presence or absence of antigen-specific antibodies but instead based on the presence or absence of antibodies that are directed against a specific epitope on a given antigen. A recently emerged example with medical importance may be provided by the differentiation of patients who developed antibodies against distinctly different epitopes on platelet factor 4 (PF4). Whereas some patients showed presence of antibodies that were directed against PF4s heparin binding site and then suffered from vaccine-induced thrombotic thrombocytopenia (VITT), other patients who suffered from related but different heparin-induced thrombocytopenia (HIT) possessed antibodies that were directed against a different but adjacently located epitope on PF4. While development of HIT normally requests administration of heparin as a medical intervention, activation of platelets to contribute to thrombocytopenia and thrombosis in VITT patients is independent from heparin treatment but was triggered by vaccination with adenoviral vector-based vaccines [37]. Knowing about the difference in the respectively recognized epitopes on PF4 enables to determine the best targeting therapy [38]. 

Blood tests, i.e., serological testing for diagnosis of allergies, are routinely used for determining presence of allergen-specific immunoglobulins [39]. According to the current routine, by chemical means, immobilized allergens are displayed to serum-borne antibodies and immune complex formation is determined by ELISA (enzyme-linked immunosorbent assay) or RAST (radioallergosorbent tests). Both ELISA and RAST request application of chemically labelled detection antibodies. On the contrary, our ITEM—SIX method neither requests antigen or antibody immobilization nor the use of chemically labeled detector molecules. Instead, ITEM—SIX simply requests to mix a patient’s or donor’s protein solution that contains an antibody of concern with a proteolysis-derived peptide mixture of the antigen of interest in which the epitope peptide is contained. Such protein/peptide solutions can be prepared with volatile buffers providing ESI-MS compatible samples. The in-solution handling that is needed for ITEM—SIX is similar to mass spectrometric epitope mapping, called epitope extraction, which forms the basis of the ITEM—ONE method developed by us [22]. 

Immunological seroconversion does typically not give rise to a single monoclonal antibody but, in fact, the immune response typically yields oligo- and/or polyclonal antibodies. Hence, the use of a monoclonal anti-Ovalbumin antibody that had been spiked into blood serum has its limitations and serves as a surrogate or mimicking experimental approximation of seroconversion. The term seroconversion remains reserved for the immuno-biological process, and the ITEM—SIX methodology will be equally applicable to real seroconverted specimen analysis. Of interest as well, although not shown in this research report, the alternative to epitope extraction, epitope excision, i.e., an immune complex is first created between the intact antigen and the antibody of concern and then is subjected to enzymatic proteolysis, is self-evident. Both epitope extraction and epitope excision have been successfully applied for analyzing sequential (linear) as well as assembled (conformational) epitopes [18], and, hence, ITEM—SIX is expected to also function in either case.

Generating immunoglobulin preparations from blood serum without the application of affinity chromatography had been developed during World War II [40,41] and was refined to fractionate serum immunoglobulins by ethanol precipitation [42,43]. Using a two-step precipitation protocol was found suitable for IgG preparation from serum and ascites fluids [44], and the first step of our IgG preparation protocol, applying octanoic acid as precipitant, followed the McKinney and Parkinson protocol. Yet, other than simply continuing with the original procedure, we replaced the second precipitating agent, ammonium sulfate, by acetone to reach an IgG preparation that is compatible with ESI-MS analysis. Acetone precipitation of proteins has been found generally applicable for concentrating proteins from aqueous solutions of all kinds [45], and protein precipitation yields were found to depend on ionic strengths of the solvents [46]. The third step of our IgG preparation protocol, i.e., filtration, followed a well-established buffer exchange procedure that has been amply applied by us in earlier ITEM projects [22,27,47].

Epitope-based serology is considered an important next step for improving diagnostic accuracy [48], which not only allows to distinguish induction of pathological processes but will enable to more precisely stratify patients for optimized and personalized treatment, as foreseen by modern precision medicine concepts [49]. To our knowledge, this is the first mass spectrometric approach that uses the mass spectrometer’s capabilities to investigate intact immune complex dissociation in the gas phase to determine—via epitope recognition—whether or not a specific antibody of interest was present in a complex IgG mixture that had been obtained from an individual’s blood serum and which, after fractionation by simple precipitation and rebuffering, was directly analyzed by nanoESI-MS. Because the complex-released epitope peptide ion signal is proof of presence of a specifically binding antibody in an IgG mixture, we termed this novel serology inspecting procedure “Intact Transition Epitope Mapping—Serology Inspection by Epitope EXtraction (ITEM—SIX)”.

## 4. Materials and Methods

### 4.1. IgG from Blood Serum

***Step 1*:** A volume of 170 µL of serum (rabbit serum; Kaninchenbetrieb Palleit, Gottin, Germany) was pipetted into a 1.5 mL reaction tube (Eppendorf SE, Hamburg, Germany). Then, 30 µL of deionized water (TKA, Milan, Italy) and 600 µL of a 60 mM sodium acetate solution (Merck, Darmstadt, Germany), prepared in de-ionized water, were added. The mixture was vortexed (Scientific Industries, Bohemia, NY, USA) for 30 s. Then, 20 µL of neat octanoic acid (Sigma-Aldrich, St. Louis, MO, USA) was slowly added. A white precipitate formed and the suspension was vortexed at room temperature for 30 min [44]. Subsequently, the suspension was centrifuged (Hettich, Tuttlingen, Germany) at 10,000× *g* and at 4 °C for 30 min to separate the white precipitate, P1, from its supernatant solution, S1. P1 was discarded. S1 (volume ca. 0.82 mL) was aspirated and filled into a 2 mL syringe (Becton Dickinson, Heidelberg, Germany), which was equipped with a PES syringe filter (0.45 µm pore size, 4 mm diameter; Agilent Technologies, SantaClara, CA, USA). S1 was filtered into a 5 mL Eppendorf tube. The filter was washed with 180 µL of deionized water and this washing solution was added to S1. The S1 solution (ca. 1 mL) may be kept at 4 °C overnight.

***Step 2*:** To solution S1 (ca. 1 mL) was added 3 mL of chilled acetone (Roth, Karlsruhe, Germany) at −20 °C. The mixture was vortexed for 1 minute (no precipitation was visible). Then, the tube was placed into the freezer (Liebherr, Ulm, Germany) and was kept at −20 °C overnight (flocculates appeared the next day). The suspension was centrifuged using the Biofuge Stratos (Thermo Fisher Scientific, Waltham, MA, USA) centrifuge at 10,000× *g* and −5 °C for 30 min to separate the white precipitate, P2, from its supernatant solution, S2. Supernatant S2 was discarded. Precipitate P2 was suspended in 100 µL of deionized water [45]. Then, the suspension was centrifuged using the Biofuge Stratos centrifuge at 10,000× *g* and 4 °C for 30 min. The supernatant solution was again discarded. This washing operation was repeated two times. Then, the precipitate P2 (ca. 3 mg) was suspended in 100 µL of 200 mM ammonium acetate (Fluka Chemika, Buchs, Switzerland), prepared in LC-MS water (Biosolve Chimie, Dieuze, France), pH 6.7, and was vortexed at room temperature overnight (max. for 10 h). The suspension was centrifuged using the Biofuge Stratos centrifuge at room temperature and at 9000 rpm for 3 min to generate supernatant S3. Then, the residual precipitate was removed by aspirating supernatant S3 (ca. 0.1 mL). Solution S3 may be kept at 4 °C overnight.

***Step 3*:** A centrifuge ultrafilter unit (Amicon Merck Millipore, Burlington, MA, USA) with 50 kDa cut-off was filled with solution S3 (ca. 0.1 mL). Then, 300 µL of 200 mM ammonium acetate, pH 6.7, were added. The solution was centrifuged (Minispin; Eppendorf SE, Hamburg, Germany) at room temperature at 9000 rpm for 5 min. The filtrate solution was discarded and the retentate solution, R1, was filled up with 200 mM ammonium acetate solution (final volume ca. 300 µL). Centrifugation, discarding of filtrate solution, and refilling were repeated eight times [22]. Then, the centrifuge ultrafilter device was mounted onto another microcentrifuge tube that was placed in the centrifuge in an upside-down position. Centrifugation (Minispin) at room temperature and at 3800 rpm was performed for 2 min. The retentate R1 (solution 0; final volume ca. 80 µL) may be kept at 4 °C for an extended period of time.

### 4.2. Anti-Ovalbumin Antibody

A volume of 50 µL of the supplier’s original anti-Ovalbumin antibody solution (6C8; protein concentration 0.98 µg/µL; abcam, Cambridge, UK) was transferred into an ultrafilter tube with 50 kDa cut-off (Amicon) and the buffer was exchanged with 0.2 M ammonium acetate, pH 6.7, using the procedure as reported above for IgG extraction (Step 3). The so prepared anti-Ovalbumin antibody solution (solution 1a; final volume 80 µL) in 0.2 M ammonium acetate, pH 6.7, had a protein concentration of 0.28 µg/µL. This solution was kept in the refrigerator for further use.

### 4.3. IgG from mAb-Spiked Serum (Surrogate Seroconversion)

To generate mAb-spiked serum, 170 µL of rabbit serum with a protein concentration of 40.2 µg/µL were added to 10 µL of anti-Ovalbumin antibody solution with 0.98 µg/µL protein concentration (original solution from supplier). Then, 175 µL of mAb-spiked serum solution were used to perform IgG extraction according to the above described IgG preparation protocol. The retentate R1 (solution 1b; final volume 62 µL) in 0.2 M ammonium acetate, pH 6.7, had a protein concentration of 1.41 µg/µL.

### 4.4. Ovalbumin Antigen and Tryptic Digestion

A volume of 10 µL of original Ovalbumin solution (P01012; Merck, Taufkirchen, Germany) with protein concentration of 9.66 µg/µL was pipetted into a 0.5 mL reaction tube and mixed with 6.8 µL of 15 M urea (BioRad, Hercules, CA, USA) dissolved in 50 mM ammonium bicarbonate solution, pH 8.0 (solution 2). To 17 µL of Ovalbumin solution (urea-containing solution 2) were added 2 µL of 100 mM DTT dissolved in 50 mM ammonium bicarbonate solution, pH 8.0, and the mixture was warmed up to 57 °C for 30 min. Then, 1.5 µL of freshly prepared 300 mM iodoacetamide (IAA; BioRad, Hercules, CA, USA) dissolved in 50 mM ammonium bicarbonate solution, pH 8.0, were added and the mixture was incubated in the dark at room temperature for 30 min. Then, 114 µL of 50 mM ammonium bicarbonate, pH 8.0, and 20 µL of Trypsin (0.1 µg/µL; Promega Corporation, Madison, WI, USA) dissolved in 3 mM TrisHCl, pH 9.3, were added. Incubation was maintained in the dark at 37 °C overnight. Final urea concentration during digestion was 0.66 M. Protein/peptide concentration was 0.63 µg/µL; final volume was 154.3 µL [35,50,51,52,53,54]. Desalting of the Ovalbumin peptide mixture and removal of urea and remaining undigested Ovalbumin was completed after digestion using a C18 OASIS cartridge (Waters Corporation, Milford, MA, USA). To a volume of 100 µL of digested Ovalbumin protein/peptide solution, pH 8.0, 900 µL of 0.1% formic acid solution prepared in LC-MS water, pH 2.6, were added. The OASIS cartridge was conditioned with 1 mL of neat acetonitrile and equilibrated with 0.1% formic acid solution, pH 2.6. After loading of the protein/peptide mixture, salt and urea were washed away first with 1 mL of 0.1% formic acid solution, pH 2.6, then with 1 mL of LC-MS water. Peptides were eluted first with 600 µL of an aqueous solution that consisted of 80% acetonitrile and 0.1% formic acid (4:1, *v*/*v*) and then with 200 µL of the same elution solvent. The eluted volumes were combined in a 1.5 mL reaction tube. Solvent was evaporated using the SpeedVac RVC 2-25 and peptides were re-dissolved in 30 µL of 0.2 M ammonium acetate prepared in LC-MS water, pH 6.7, to yield the digested Ovalbumin peptide solution (solution 2′) with final peptide concentration of 0.63 µg/µL. 

### 4.5. SDS-PAGE Analysis

A volume of IgG-containing solution (solutions 0, 1a, or 1b) that contained between 0.5 µg and 1 µg of protein was filled up with deionized water to reach a total volume of 16 µL. Then, 4 µL of non-reducing sample buffer were added. Alternatively, 4 µL of reducing sample buffer were added to the diluted IgG solutions and these solutions were mixed and heated (HLC Heater, Labor Consult, Bovenden, Germany) at 95 °C for 5 min. To check the tryptic digestion yield, an SDS-PAGE run was performed using a 1.2 µL aliquot from the Ovalbumin digestion mixture (solution 2′) and 3.2 µL of Ovalbumin solution 0.24 µg/µL, prepared by dilution of 2 µL of Ovalbumin solution 9.66 µg/µL with 78 µL of 50 mM ammonium bicarbonate, pH 8.0. SDS-PAGE analysis was performed as described elsewhere (see details in Supplementary Materials) [55,56].

### 4.6. Offline nanoESI-MS Analysis of Ovalbumin Peptides

A volume of 1 µL of tryptic Ovalbumin peptide mixture (solution 2′) was diluted with 3 µL of 0.2 M ammonium acetate, pH 6.7, to yield a final peptide concentration of 0.28 µg/µL. 2.5 µL of this diluted tryptic Ovalbumin peptide mixture was filled into a gold-coated nanospray needle for offline nanoESI-MS analysis [57]. Offline nanoESI-MS analysis at the Q-ToF II instrument was performed applying a capillary voltage of 1 kV. Cone voltage was set to 130 V. Extractor voltage was 3 V. RF Lens was set to 1.2 V. Source temperature was 40 °C. MCP detector voltage was 1950 V. The pusher was set to 124 µs. Inlet vacuum was 1.5 × 10^−1^ mbar. Analyzer Penning was 3 × 10^−5^ mbar. The ToF analyzer vacuum was 4.5 × 10^−7^ mbar. The nitrogen sheath gas flow was set to 4 psi. Mass spectra were recorded from *m*/*z* 50 to *m*/*z* 3000 for 5 min. Obtained spectra were smoothed using the method “mean”. Spectra were recorded using the MassLynx 4.0 data system from Waters (Manchester, UK). Raw data were exported and graphic files (CorelDraw 17.0 software package) were saved on computer drives. Obtained raw data were used for peptide assignments by the PLGS 3.0 software.

### 4.7. Offline nanoESI-MS Analysis of Intact IgG

At this point, 2.5 µL of the retentate R1 (solution 0) or 2.5 µL of anti-Ovalbumin antibody (solution 1a) were transferred into a gold-coated capillary needle and analyzed by offline nanoESI-MS using the Q-TOF II mass spectrometer (Waters, Manchester, UK) [47]. Likewise, 2.5 µL of IgG from converted serum (solution 1b) were diluted with 29 µL of 0.2 M ammonium acetate, pH 6.7, to yield a protein concentration of 0.17 µg/µL. Then, 2.5 µL of this diluted solution 1b were transferred into a gold-coated capillary needle. The capillary needle voltage was set to 1.4 kV, cone voltage to 130 V, extractor voltage to 3 V, RF lens to 1.2 V. Source temperature was 40 °C and the nitrogen sheath gas flow was set to 4 psi. The multichannel plate detector voltage was set to 1950 V and pusher time to 124 µs. Inlet vacuum was 1.5 × 10^−1^ mbar; quadrupole analyzer vacuum was 3.9 × 10^−5^ mbar (softer desolvation conditions); the TOF analyzer vacuum was 3.2 × 10^−7^ mbar [18]. Alternatively, quadrupole analyzer vacuum of 3.2 × 10^−5^ mbar (harsher desolvation conditions) was applied. The mass spectra were collected from *m*/*z* 500 to *m*/*z* 7500 for ca. 5 min each. Spectra were recorded using the MassLynx 4.0 data system from Waters (Manchester, UK). Obtained mass spectra were smoothed in 3 cycles with a window of 5 for low mass range spectra and with 50 cycles with a window of 100 for high mass range spectra, respectively, using the Savitzky–Golay algorithm. The MassLynx 4.0 software package was used for data analysis and spectral image preparation in conjunction with the CorelDraw 2017 software package [26].

### 4.8. ITEM—ONE Epitope Mapping and Negative Control

A volume of 10 µL of anti-Ovalbumin antibody solution (solution 1a) with a protein concentration of 0.28 µg/µL; 1.9 pmol/µL) was mixed with 3.1 µL of peptide mixture from digested Ovalbumin solution (solution 2′) with a peptide concentration of 0.055 µg/µL. This Ovalbumin peptide solution was obtained by dilution of 1 µL of digested Ovalbumin solution (peptide concentration 0.28 µg/µL) with 4 µL of 0.2 M ammonium acetate, pH 6.7. The mixture (solution 3a) has a final antibody concentration of 0.21 µg/µL (1.4 pmol/µL) and a final peptide concentration of 0.013 µg/µL (32 pmol/µL). After mixing, solution 3a was incubated at room temperature for at least 1.5 h. A volume of 2.5 µL of solution 3a was filled into a gold-coated nanospray capillary needle. Offline nanoESI-MS ITEM—ONE analysis was performed as described [22]. Settings of capillary needle voltage were 1.4 kV, cone voltage 130 V, extractor voltage 3 V, RF lens 1.2 V. Source temperature was set to 80 °C and the nitrogen sheath gas flow was set to 4 psi. Multichannel plate detector voltage was set to 1950 V and pusher time to 124 µs. Inlet vacuum was 1.5 × 10^−1^ mbar, quadrupole analyzer vacuum was 3.5 × 10^−5^ mbar, the ToF analyzer vacuum was 4.5 × 10^−7^ mbar. Transmission of ions below *m*/*z* 1450 was completely blocked via respective quadrupole settings: M1 = 2000, dwell time, and ramp time 25%; M2 = 2000, dwell time, and ramp time 25%; and M3 = 2000. Mass spectra were recorded in ranges from *m*/*z* 200 to *m*/*z* 8000. Collision gas pressure was set to 4 psi and collision cell voltage difference initially was set to 3 V. After 1.5 min of recording, the collision cell voltage difference was increased to 20 V. Recordings lasted again for 1.5 min each using the MassLynx 4.0 data system (Waters, Manchester, UK). Obtained mass spectra were smoothed in 3 cycles with a window of 5 for low mass range spectra and with 50 cycles with a window of 100 for high mass range spectra, respectively, using the Savitzky–Golay algorithm. The MassLynx 4.0 software package was also used for data analysis and spectral image preparation in conjunction with the CorelDraw 17.0 software package.

### 4.9. ITEM—SIX Analysis and Negative Control

A volume of 5 µL of digested Ovalbumin solution (solution 2′) with 0.32 µg/µL protein concentration was obtained by dilution with 0.2 M ammonium acetate, pH 6.7. To this diluted Ovalbumin peptide solution (solution 2′) were added 4 µL of IgG extracted from converted rabbit serum (solution 1b; protein concentration 1.41 µg/µL). The mixture (solution 3b) has a final volume of 9 µL, a final antibody concentration of 0.63 µg/µL (4.2 pmol/µL), and a final Ovalbumin peptide concentration of 0.18 µg/µL. Solution 3b was incubated at room temperature for 1.5 h. A volume of 2.5 µL of solution 3b was filled into a gold-coated nanospray capillary needle for offline nanoESI-MS ITEM—SIX experiments.

IgG extracted from rabbit serum (solution 0) served as negative control and was diluted with 0.2 M ammonium acetate, pH 6.7, to a protein concentration of 0.23 µg/µL (1.6 pmol/µL). A volume of 2.5 µL of digested Ovalbumin peptide solution (solution 2′) with peptide concentration of 0.055 µg/µL (13.7 pmol/µL) was added to 10 µL of diluted IgG from rabbit serum (solution 0) to generate solution 3c. The final volume of solution 3c was 12.5 µL, final IgG concentration 0.19 µg/µL (1.2 pmol/µL), and final Ovalbumin peptide concentration 0.011 µg/µL (34.0 pmol/µL). After mixing, solution 3c was incubated at room temperature for at least 1.5 h. A volume of 2.5 µL of solution 3c was filled into a gold-coated nanospray capillary needle for offline nanoESI-MS ITEM—SIX experiments.

Offline nanoESI-MS ITEM—SIX analysis was performed as described above for ITEM—ONE, but, this time, the applied capillary needle voltage varied between 1.2 and 1.8 kV and the quadrupole analyzer vacuum was varied between 3.5 × 10^−5^ mbar (soft desolvation conditions) and 4.4 × 10^−5^ mbar (inefficient desolvation conditions). Obtained mass spectra were smoothed in 3 cycles with a window of 5 for low mass range spectra and with 50 cycles with a window of 100 for high mass range spectra, respectively, using the Savitzky–Golay algorithm. The mass spectrometry data have been deposited to the ProteomeXchange Consortium via the PRIDE [58] partner repository with the dataset identifier PXD040754.

### 4.10. Western Blot Analysis

Western blot analysis was performed as described elsewhere (see details in Supplementary Materials) [56]. The blocking buffer for primary antibody solutions was prepared by adding 0.1% (*v*/*v*) Tween-20 to a 1:2 dilution of Intercept Blocking buffer (LI-COR Biosciences, Lincoln, NE, USA) with PBS buffer. First, 35 µL of anti-Ovalbumin antibody (solution 1a with 0.017 µg/µL) were added to 3 mL blocking buffer and used as primary antibody solution. Second, 40 µL of R1 (solution 0), i.e., IgG extracted from rabbit blood serum, were used and mixed with 3 mL blocking buffer solution to obtain the negative control solution.

## 5. Conclusions

The unique combination of (i) targeted pre-analytical methods for rapid analyte preparation and (ii) advanced analytical procedures with high sensitivity and low sample consumption paves the way for cutting-edge serological inspection in an unprecedented way. ITEM—SIX provides the means to precisely learn about presence or absence of an antibody of concern in an individual patient’s/donor’s blood serum with neither the need for chemical immobilization of the antigen nor the use of a labeled reporter molecule for detection. ITEM—SIX exploits the binding motif recognition capability of an antibody, which leads to epitope-based stratification of patient cohorts with unsurpassed precision.

## Data Availability

The mass spectrometry data have been deposited to the ProteomeXchange Consortium via the PRIDE [58] repository with the dataset identifier PXD040754.

## Supplementary Materials

The following supporting information can be downloaded at: https://www.mdpi.com/article/10.3390/molecules28073092/s1, Figure S1: SDS-PAGE analysis of rabbit blood serum proteins and therefrom isolated IgG antibody fraction.; Figure S2: SDS-PAGE analysis of Ovalbumin antigen and digestion control.; Figure S3: Western blot analysis of Ovalbumin antigen.; Figure S4: ITEM—ONE analysis.; Figure S5: ITEM—ONE analysis, negative control.; Figure S6: ITEM—ONE analysis, negative control.; Figure S7: NanoESI tandem mass spectrum of Ovalbumin epitope peptide precursor ion (*m*/*z* 1687.71) from tryptic digestion (solution 2′).; Figure S8: ITEM—SIX analysis.; Figure S9: ITEM—SIX analysis, negative control.; Figure S10: ITEM—SIX analysis, negative control.; Table S1: Multiply charged ion signals, charge states, and experimental molecular masses of IgG, anti-Ovalbumin antibody, and immune complex.; Table S2: Assigned peptide ion signals from in-gel tryptic digestion of ovalbumin from chicken egg white.; Table S3: Unassigned peptide ion signals from in-gel tryptic digestion of ovalbumin from chicken egg white.
