# Supplementary material for: Intact Transition Epitope Mapping—Serological Inspection by Epitope EXtraction (ITEM—SIX)
Source: Molecules. 2023 Mar 30;28(7):3092. doi: 10.3390/molecules28073092 (PMC10096252; doi:10.3390/molecules28073092)
Supplement: Supplementary file 1 [file molecules-28-03092-s001.zip › molecules-2268454-supplementary.pdf]

Supplement

Intact Transition Epitope Mapping—Serological Inspection by Epitope EXtraction (ITEM—SIX) †

Agatino Zammataro [1,2], Cornelia Koy [1], Manuela Ruß [1], Claudia Röwer [1] and Michael O. Glocker [1,*]

[1] Proteome Center Rostock, University Medicine Rostock and University of Rostock, Schillingallee 69, 18059 Rostock, Germany

[2] Department of Chemical Sciences, University of Catania, Viale Andrea Doria 6, 95125 Catania, Italy

* Correspondence: michael.glocker@uni-rostock.de; Tel.: +49-381-494-4930

† In memoriam of Prof. Dr. Dr. h.c. Michael Przybylski who suddenly and unexpectedly passed away in February 2023.

Abstract: Precision medicine requests accurate serological inspections to precisely stratify patients for targeted treatment. Intact transition epitope mapping analysis proved surrogate seroconversion of a model organism's serum when spiked with a monoclonal murine anti-Ovalbumin antibody (mAb) with epitope resolution. Isolation of the IgG fraction from blood serum applied two consecutive protein precipitation steps followed by ultrafiltration and resulted in an ESI-MS analysis-ready IgG preparation. For epitope mapping by epitope extraction, the Ovalbumin antigen was digested with trypsin. After desalting, the peptide mixture was added to the ESI-MS-ready IgG preparation from mAb-spiked serum and the solution was incubated to form an immune complex between the Ovalbumin-derived epitope peptide and the anti-Ovalbumin mAb. Then, the entire mixture of proteins and peptides was directly electrosprayed. Sorting of ions in the mass spectrometer's gas phase, dissociation of the immune complex ions by collision-induced dissociation, and recording of the epitope peptide ion that had been released from the immune complex proved the presence of the anti-Ovalbumin mAb in serum. Mass determination of the complex-released epitope peptide ion with isotope resolution is highly accurate, guaranteeing high specificity of this novel analysis approach, which is termed Intact Transition Epitope Mapping—Serological Inspections by Epitope EXtraction (ITEM—SIX).

Keywords: blood serum; surrogate seroconversion; immune complex analysis; epitope mapping; nanoESI mass spectrometry

Supplemental Materials and Methods

SDS-PAGE Analysis

The pre-cast SDS gel (82 mm × 68 mm × 1 mm; NuPAGE Novex 12% Bis-Tris Gel; Invitrogen, Carlsbad, CA, USA) was placed in the electrophoresis chamber (Hoefer DALT Vertical Electrophoresis System; GE healthcare/Amersham Biosciences, Freiburg, Germany) and the chamber was filled with running buffer (104.6 g of 3-N-Morpholino propanesulfonic acid (Serva Electrophoresis, Heidelberg, Germany), 60.6 g of Tris (tris(hydroxymethyl)amin methane (Roth, Karlsruhe, Germany), 10 g of sodium dodecyl sulfate (Serva Electrophoresis), and 3.8 g of Titriplex III (ethylenedinitrilotetraacetic acid disodium salt dehydrate, Merck, Darmstadt, Germany) in 500 mL of deionized water and was diluted 1:20 with deionized water. Into each of the gel's pockets were filled either 20 µL of protein-containing solution or 3 µL of prestained protein marker mix (Thermo Fisher Scientific; Waltham, MA, USA). Protein solutions were prepared with non-reducing sample buffer (6.25 mL of 1 M TrisHCl, pH 6.8, 2 g SDS, 0.08 g bromophenol blue (Merck), 10 mL of glycerol (Merck), and 3.75 mL of deionized water) or reducing sample buffer (6.25 mL of 1 M TrisHCl, pH 6.8, 2 g SDS, 0.08 g bromophenol blue, 1 g dithiothreitol (Serva Electrophoresis), 10 mL glycerol, and 3.75 mL of deionized water). Then, electrophoresis was performed at 200 V and 150 mA for 45 min. After separation, the gel was placed in 50 mL fixation solution (50% ethanol (Zentralapotheke Universitätsmedizin Rostock, Rostock, Germany) and 10% acetic acid (Baker, Deventer, The Netherlands) in deionized water) and shaken at room temperature for 1 h.

The proteins were stained by adding 50 mL Coomassie brilliant blue staining solution, shaking at room temperature overnight. Coomassie brilliant blue G250 (Serva Electrophoresis) staining solution was prepared with 100 g of $Al_2(SO_4)_3$ (Sigma-Aldrich, St. Louis, Missouri, USA), which was solubilized in 1.5 l of deionized water. Then, 200 mL of ethanol and 0.4 g of Coomassie brilliant blue G250 were added. Then, 46 mL of 85% ortho-H_3PO_4 (Merck) were added. The solution was filled up with deionized water until a final volume of 2 l. Afterwards, background was destained 4 times for 20 min each, shaking with 50 mL 10% (*v*/*v*) of ethanol and 2.3% of 85% H_3PO_4 in deionized water. The gels were scanned using the ScanMaker 1000XL (Microtek, Hsinchu City, Taiwan) and gel images were subjected to image analysis and documentation using the CorelDraw 17.0 software package.

To stain the proteins in the gel by silver staining (after Coomassie staining), the gel was placed in fixation solution (ca. 50 mL) and shaken at room temperature overnight. Then, the gel was placed in 50% ethanol solution (ca. 50 mL) and shaken at room temperature for 20 min. This operation was repeated 2 times. Then, the gel was placed in 50 mL Sensitizing solution (100 mg of $Na_2S_2O_3 \cdot \bullet 5H_2O$ (Merck) in 500 mL deionized water) and was shaken at room temperature for 2 min. Then, the gel was placed in deionized water (ca. 50 mL) and shaken at room temperature for 1 min. This operation was repeated. Then, 100 mL of silver nitrate solution (1 g $AgNO_3$ (Merck) in 500 mL deionized water) were mixed with 75 µL of 37% formaldehyde (Merck) and the gel was submerged in this solution (ca. 50 mL) and shaken in the dark at room temperature for 20 min. Then, the gel was placed in deionized water (ca. 50 mL) and shaken at room temperature for 1 min. Then, the gel was placed in 50 mL Developing solution (25 mL of Sensitizing solution with 60 g of Na_2CO_3 (Merck) and 975 mL of deionized water) to which had been added 25 µL of formaldehyde. The gel was shaken at room temperature until developed (ca. 15 s). Next, the gel was placed in 50 mL Stop solution (1.46 g of Titriplex III in 100 mL deionized water) and shaken at room temperature for 10 min. At last, the gel was scanned using the ScanMaker 1000XL scanner; tif-files were subjected to image analysis and documentation using the CorelDraw 17.0 software package.

Western Blot Analysis

An Immobilon-FL PVDF membrane (0.45 µm; Millipore, Burlington, MA, USA) was cut according to the size of the gel (82 mm × 68 mm) and was moistened with ca. 10 mL iso-propanol (Roth) until fully soaked. The PVDF membrane was shaken in deionized water (ca. 50 mL) and stored in LT-buffer, pH 10.4, (1.51 g TRIS, 100 mL methanol in 400 mL deionized water) until blot preparation. Next, 18 filter paper pieces (Gel-Blotting Paper; Schleicher&Schuell, Keene, NH, USA) with the dimensions of 82 mm x 68 mm were prepared. After gel electrophoresis, the gel was stored in ε-buffer, pH 9.4 (2.62 g aminocaproic acid (Merck), 1.51 g Tris, 100 mL methanol (Roth) in 400 mL deionized water) for ca. 30 min. The floor of the Western blot equipment (Semi-Dry Blotter "Pegasus"; PHASE, Lübeck, Germany) was moistened with ε-buffer and 9 layers of filter papers that were soaked in ε-buffer were placed on the floor of the Western blot chamber, followed by the gel, the wetted PVDF membrane, 3 layers of filter paper that were soaked in LT buffer, and 6 layers of filter paper that were impregnated in HT buffer, pH 10.4 (18.16 g TrisHCl, 100 mL methanol in 400 mL deionized water). The lid of the blot chamber was moistened with HT buffer and, after closing, the chamber was burdened with an extra weight of approx. 3 kg. The proteins were blotted onto the PVDF membrane by semi-dry blotting with constant electric current of 64 mA for 45 min. After electroblotting, the PVDF membrane was blocked with 10 mL blocking buffer (Intercept Blocking buffer (LI-COR Biosciences, Lincoln, NE, USA) diluted 1:2 with PBS (140 mM sodium chloride (J.T. Baker, Deventer, The Netherlands), 10 mM di-Sodium hydrogen phosphate dehydrate (Merck), 2.7 mM potassium chloride (Merck), 1.8 mM potassium dihydrogen phosphate (Merck)) shaking at room temperature for 1 h. Afterwards, the PVDF membrane was cut into strips. The first strip contained the prestained protein marker proteins (lane 1 in Figure S3) and Ovalbumin (lane 2 in Figure S3); the second strip contained Ovalbumin alone (lane 3 in Figure S3). The first strip was incubated with 35 µL anti-Ovalbumin antibody (solution 1a with 0.017 µg/µL) in 3 mL blocking buffer with 0.1% Tween 20. The second strip was incubated with 40 µL IgG preparation from serum (R1, solution 0) in 3 mL blocking buffer with 0.1% Tween 20. Incubations of the PVDF membranes were performed on a shaker at 4 °C overnight. After discarding the primary antibody solutions, the PVDF membrane strips were washed separately four times with ca. 10 mL of washing buffer (PBS with 0.1% Tween 20) for 5 min each. Then, each strip was incubated separately with 3 mL of the secondary antibody solution (1 µL of antiMouse-IgG antibody from goat coupled with IRDye® 800 CW (Li-COR Biosciences, Lincoln, NE, USA) in 10 mL blocking buffer) for one hour at room temperature. After discarding the secondary antibody solutions, the PVDF membrane strips were washed with ca. 10 mL of washing buffer four times and for 5 min each. Images were obtained using the Odyssey DLx Imaging system (Li-COR Biosciences; Bad Homburg vor der Höhe, Germany) with an excitation wavelength of 800 nm. The scans provided tif-files; image analysis and documentation were conducted using the CorelDraw 17.0 software package.

Supplemental Tables

Table S1. Multiply charged ion signals, charge states, and experimental molecular masses of IgG, anti-Ovalbumin antibody, and immune complex.

m/z	z	Mass (exp.) [Da]	Average Mass (std. dev.) [Da]
**1) IgG Preparation** [a]			
5353.3	28	149,962	
5551.4	27	148,321	
5767.2	26	149,921	
6023.3	25	150,558	
6284.6	24	150,805	
6562.5	23	150,914	**150,080 (±957)**
**2) Anti-Ovalbumin Antibody** [b]			
5928.0	25	148,176	
6183.2	24	148,374	
6447.8	23	148,277	
6741.9	22	148,300	
7073.4	21	148,520	**148,329 (±128)**
**3) Immune Complex** [c]			
5994.4	25	149,835	
6240.0	24	149,736	
6512.6	23	149,766	
6809.4	22	149,784	
7138.9	21	149,895	**149,803 (±63)**

[a] cf. Figure 1A.

[b] cf. Figure 2A.

[c] cf. Figure S4A.

Table S2. Assigned peptide ion signals from in-gel tryptic digestion of ovalbumin from chicken egg white.

Sequence Range	*m*/*z* exp.	mass (calcd)	*z*	Amino Acid Sequence
1–16 [a)]	1807.44	1807.80	1	ac-GSIGAASMEFCFDVFK
1–16 [a)]	904.33	904.41	2	ac-GSIGAASMEFCFDVFK
47–55	531.12	531.78	2	DSTRTQINK
59–84	1411.67	1411.18	2	FDKLPGFGDSIEAQCGTSVNVHSSLR
59–84 [b)]	1451.19	1451.17	2	FDKLPGFGDSI-EAQCGTpSVNVHSSLR
59–84 [b)]	967.67	967.78	3	FDKLPGFGDSI-EAQCGTpSVNVHSSLR
62–84 [b)]	1256.06	1256.07	2	LPGFGDpSIEAQCGTSVNVHSSLR
62–84 [b)]	837.66	837.72	3	LPGFGDpSIEAQCGTSVNVHSSLR
85–104	1141.02	1141.09	2	DILNQITKPNDVYSFSLASR
105–110	780.37	780.39	1	LYAEER
111–122	1522.80	1522.80	1	YPILPEYLQCVK
111–122	761.89	761.90	2	YPILPEYLQCVK
123–126	580.28	580.31	1	ELYR
127–142	1687.71	1687.84	1	GGLEPINFQTAADQAR
127–142	844.37	844.42	2	GGLEPINFQTAADQAR
143–158	1859.44	1858.97	1	ELINSWVESQTNGIIR
143–158	930.39	929.99	2	ELINSWVESQTNGIIR
182–186	632.37	632.34	1	GLWEK
182–199	2168.71	2169.04	1	GLWEKAFKDEDTQAMPFR
182–199	1085.45	1085.03	2	GLWEKAFKDEDTQAMPFR
187–199	1555.72	1555.72	1	AFKDEDTQAMPFR
187–199	778.33	778.36	2	AFKDEDTQAMPFR
219–226	822.36	822.40	1	VASMASEK
264–276	1581.69	1581.72	1	LTEWTSSNVMEER
264–276	791.33	791.36	2	LTEWTSSNVMEER
264–277	855.36	855.41	2	LTEWTSSNVMEERK
280–284	647.41	647.39	1	VYLPR
323–339	1773.59	1773.90	1	ISQAVHAAHAEINEAGR
323–339	887.38	887.45	2	ISQAVHAAHAEINEAGR
323–339	591.94	591.97	3	ISQAVHAAHAEINEAGR
340–359	2009.00	2008.95	1	EVVGSAEAGVDAASVSEEFR
340–359	1004.87	1004.98	2	EVVGSAEAGVDAASVSEEFR
340–359 [b)]	2088.75	2088.91	1	EVVGpSAEAGVDAASVSEEFR
340–359 [b)]	1044.86	1044.96	2	EVVGpSAEAGVDAASVSEEFR
360–369	1247.63	1247.62	1	ADHPFLFCIK
360–369	624.33	624.32	2	ADHPFLFCIK
370–381 [c)]	664.35	664.37	2	HIATNAVLFFGR
370–381	673.39	673.37	2	HIATNAVLFFGR

[a)] acetylated N-terminus.

[b)] phosphorylated serine.

[c)] loss of water.

Table S3. Unassigned peptide ion signals from in-gel tryptic digestion of ovalbumin from chicken egg white.

Singly Protonated (m/z)	Doubly Protonated (m/z)	Triply Protonated (m/z)
548.22	754.35	972.99
1613.63	767.36	974.99
1638.65	786.33	980.33
1823.42	863.32	1128.42
1891.34	938.37	1307.33
	941.36	1326.70
	946.37	1658.65
	949.36	1719.57
	1055.86	
	1186.58	
	1289.07	
	1320.62	
	1406.07	
	1458.64	
	1462.20	
	1466.68	
	1470.18	
	1507.67	
	1609.05	
	1692.08	
	1959.72	
	2109.66	

Supplemental Figures

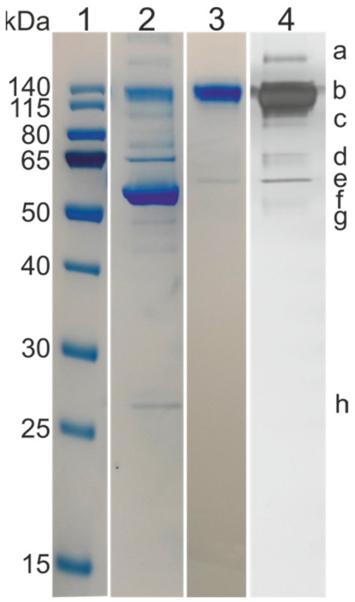

Figure S1. SDS-PAGE analysis of rabbit blood serum proteins and therefrom isolated IgG antibody fraction. Lane 1: molecular mass marker (PageRuler Prestained Protein Ladder). Apparent molecular masses are shown at the left. Lane 2: rabbit serum. Lanes 3 and 4: from rabbit serum, isolated IgG. 1 μg of protein was loaded per lane. Proteins in lanes 1–3 were stained with colloidal Coomassie Brilliant Blue and proteins in lane 4 were silver-stained. Bands are alphabetically labelled on the right. Protein assignments: band a: unknown; band b: IgG; band c: haptoglobin; band d: serotransferrin; band e: serum albumin; band f: transthyretin; band g: unknown; band h: apolipoprotein A1.

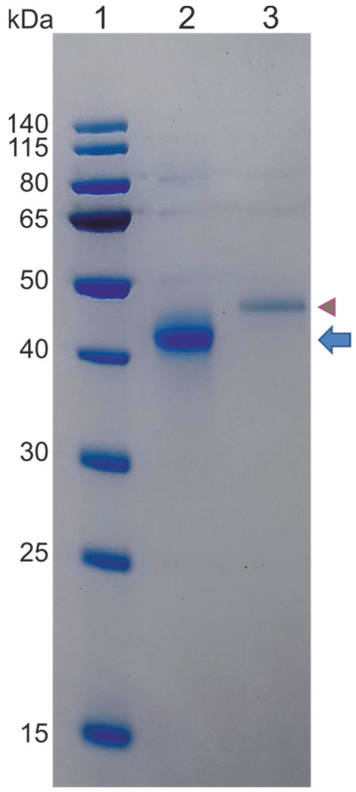

Figure S2. SDS-PAGE analysis of Ovalbumin antigen and digestion control. Lane 1: molecular mass marker (PageRuler Prestained Protein Ladder). Apparent molecular masses are shown at the left. Lane 2: commercial Ovalbumin (blue arrow). Lane 3: reduced and alkylated Ovalbumin (purple arrow head) after tryptic digestion. 0.77 µg Ovalbumin/digested Ovalbumin was loaded per lane. Proteins were stained with colloidal Coomassie Brilliant Blue.

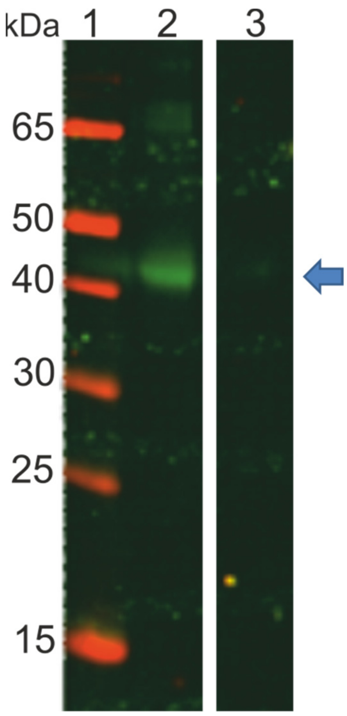

Figure S3. Western blot analysis of Ovalbumin antigen. Lane 1: molecular mass marker (PageRuler Prestained Protein Ladder). Apparent molecular masses are shown at the left. Lanes 2 and 3: commercial Ovalbumin; 1.1 µg Ovalbumin was loaded per lane. Anti-Ovalbumin antibody (solution 1a) was used as primary antibody in lane 2. IgG mixture prepared from rabbit serum (solution 0) was used as primary antibody in lane 3. Polyclonal anti-mouse IgG antibody from goat labelled with IRDye® 800 CW was used as secondary antibody. The blue arrow on the right points to the location of the (decorated) Ovalbumin antigen. Blot images have been cropped above 80 kDa and below 10 kDa, respectively.

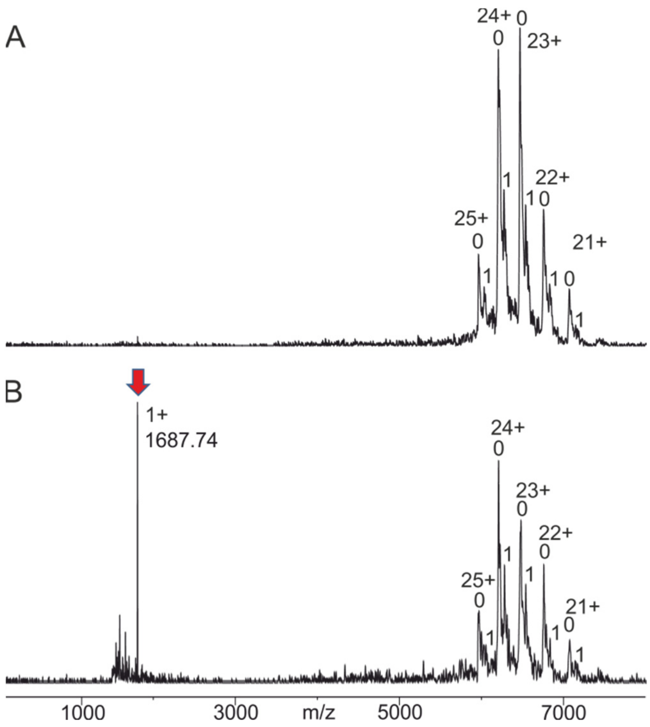

Figure S4. ITEM—ONE analysis. Nano-ESI mass spectra of tryptic Ovalbumin peptide mixture (solution 2′) plus anti-Ovalbumin antibody (solution 1a) and formed immune complex (solution 3a). **A:** ΔCV: 3 V. **B:** ΔCV: 20 V. Ion transmission below m/z 1450 is completely suppressed. Charge states (right ion series) are provided for the multiply charged ion signals of the antibody (0) and the immune complex (1, antibody plus one peptide). Charge state and m/z value for complex-released peptide ion signal are provided on the left. Protein concentration is 0.22 µg/µL. 2.5 µL were loaded. Solvent: 200 mM ammonium acetate, pH 6.7. Quadrupole vacuum: 3.5×10^{-5} mbar. Recording time per spectrum is 1 min.

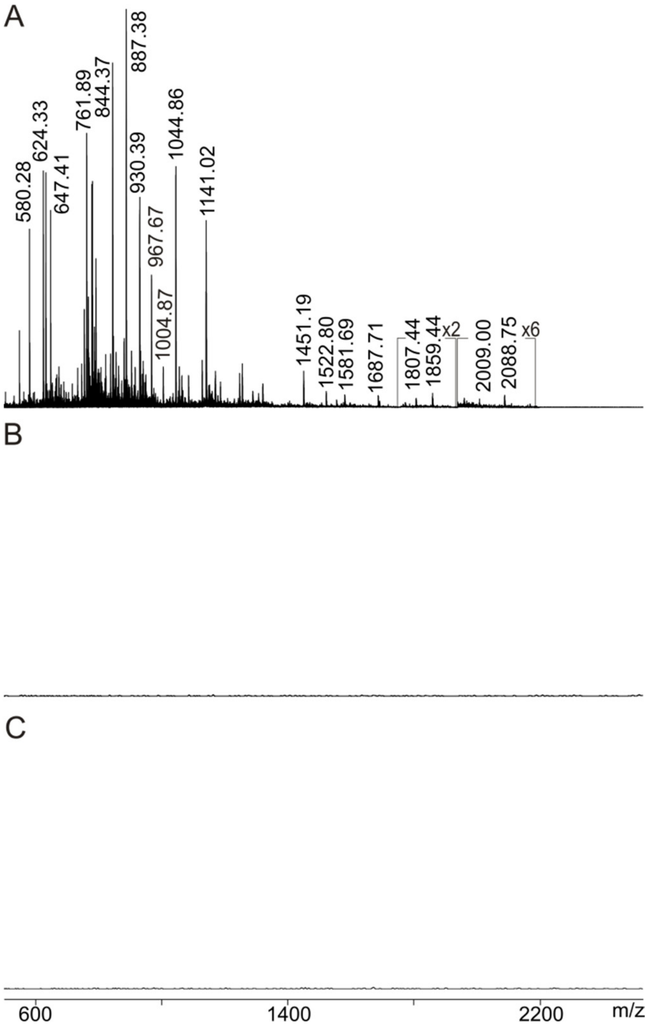

Figure S5. ITEM—ONE analysis, negative control. **A:** NanoESI mass spectrum of ions from Ovalbumin upon tryptic digestion (solution 2´). Ion intensity multiplication factors are provided. **B** and **C:** Nano-ESI mass spectra (solution 3c) of tryptic Ovalbumin peptide mixture (solution 2´) plus IgG mixture prepared from rabbit serum (solution 0). **B:** ΔCV: 3 V. **C:** ΔCV: 20 V. Ion transmission below m/z 1450 is completely suppressed. Selected m/z values are provided (cf. Table S2). Protein concentration is 0.20 µg/µL. 2.5 µL were loaded. Solvent: 200 mM ammonium acetate, pH 6.7. Recording time per spectrum is 1 min.

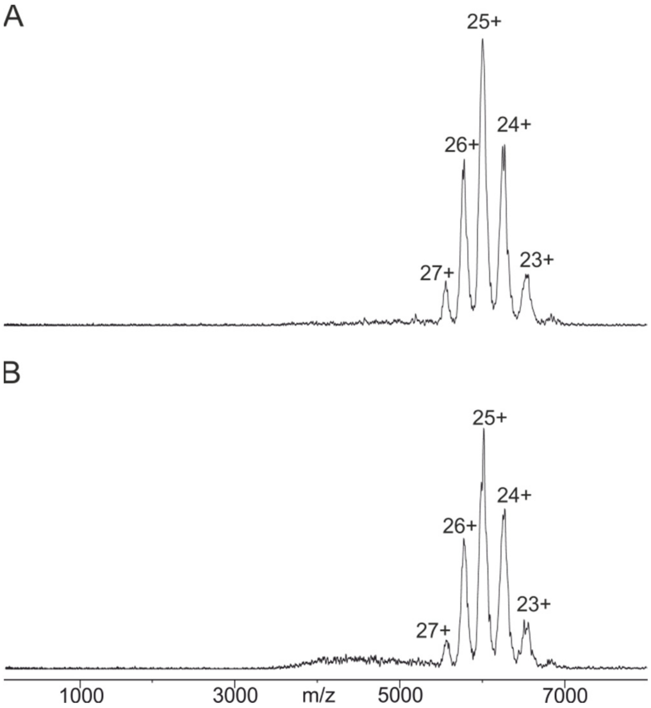

Figure S6. ITEM—ONE analysis, negative control. Nano-ESI mass spectra (solution 3c) of tryptic Ovalbumin peptide mixture (solution 2´) plus IgG mixture prepared from rabbit serum (solution 0) **A:** ΔCV: 3 V. **B:** ΔCV: 20 V. Ion transmission below m/z 1450 is completely suppressed. Charge states are provided for the multiply charged ion signals of the IgG mixture. Protein concentration is 0.20 μg/μL. 2.5 μL were loaded. Solvent: 200 mM ammonium acetate, pH 6.7. Quadrupole vacuum: 3.5 × 10^{-5} mbar. Recording time per spectrum is 1 min.

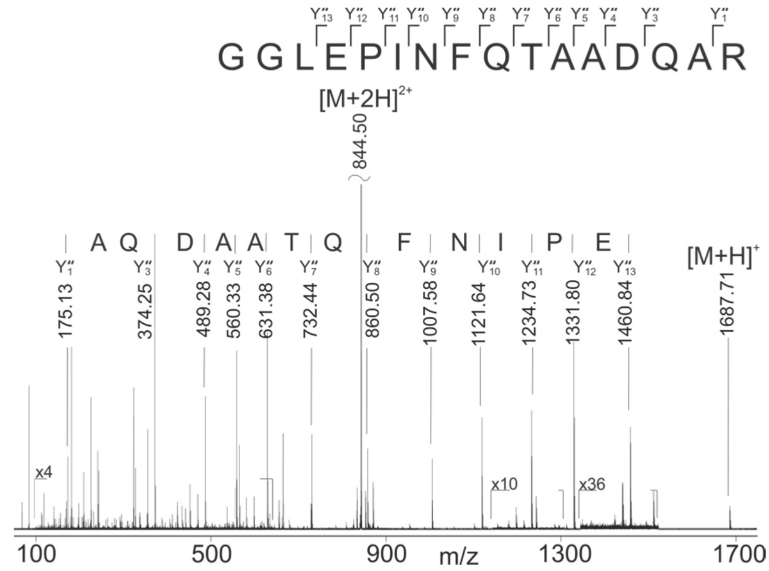

Figure S7. NanoESI tandem mass spectrum of Ovalbumin epitope peptide precursor ion (m/z 1687.71) from tryptic digestion (solution 2′). Selected and assigned Y-type fragment ion signals are labelled. Ion intensity multiplication factors are provided. Peptide mixture concentration is 0.28 µg/µL. 2.5 µL were loaded. Solvent: 200 mM ammonium acetate, pH 6.7. Recording time is 1 min. The amino acid sequence of the precursor peptide is shown on top.

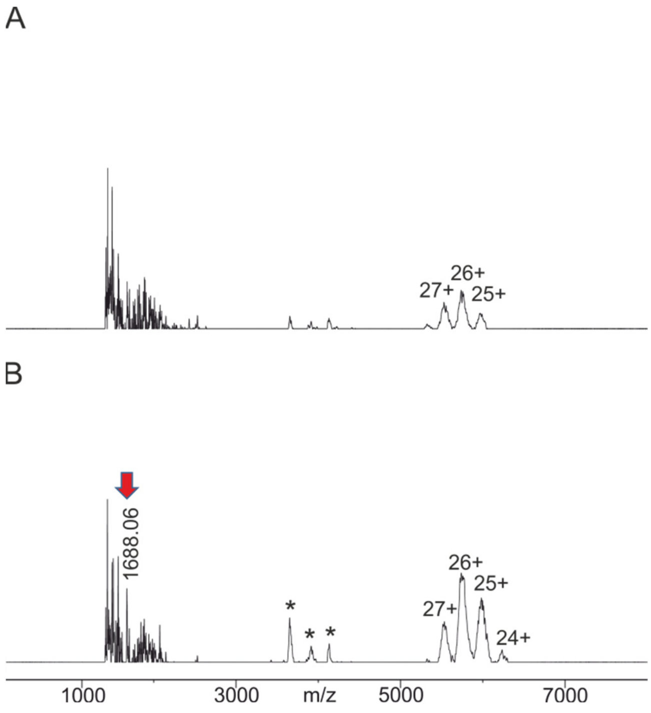

Figure S8. ITEM—SIX analysis. Nano-ESI mass spectra of tryptic Ovalbumin peptide mixture (solution 2′) plus IgG preparation with anti-Ovalbumin mAb-spiked serum (solution 1b) and formed immune complex (solution 3b). **A:** ΔCV: 3 V. **B:** ΔCV: 20 V. Ion transmission below m/z 1450 is completely suppressed. Charge states (right ion series) are provided for the multiply charged ion signals of the IgG mixture. M/z value for the complex-released peptide ion signal is provided on the left (red arrow). Asterisks mark multiply charged ion signals from serum albumin. Protein concentration is 0.81 µg/µL. 2.5 µL were loaded. Solvent: 200 mM ammonium acetate, pH 6.7. Quadrupole vacuum: 4.4×10^{-5} mbar. Recording time per spectrum is 1 min. 40% baseline subtraction.

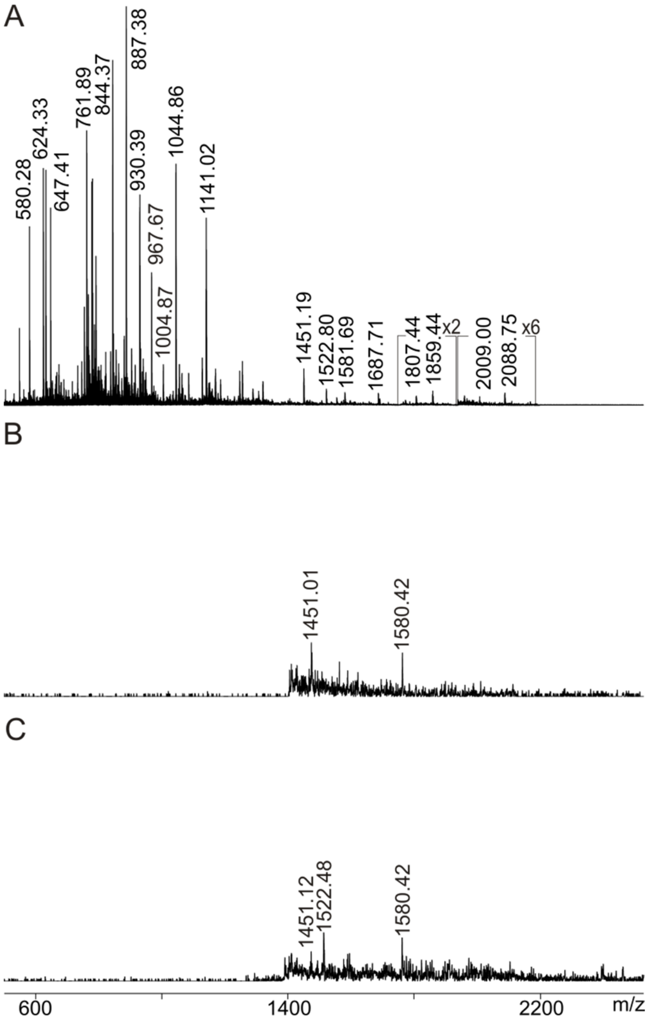

Figure S9. ITEM—SIX analysis, negative control. **A:** NanoESI mass spectrum (solution 3c) of ions from Ovalbumin upon tryptic digestion (solution 2′). **B** and **C:** Nano-ESI mass spectra of tryptic Ovalbumin peptide mixture (solution 2′) plus IgG mixture prepared from rabbit serum (solution 0). **B:** ΔCV: 3 V. **C:** ΔCV: 20 V. Ion transmission below m/z 1450 is completely suppressed. Selected m/z values are provided (cf. Table S2). Protein concentration is 0.20 µg/µL. 2.5 µL were loaded. Solvent: 200 mM ammonium acetate, pH 6.7. Recording time per spectrum is 1 min. 30% baseline subtraction.

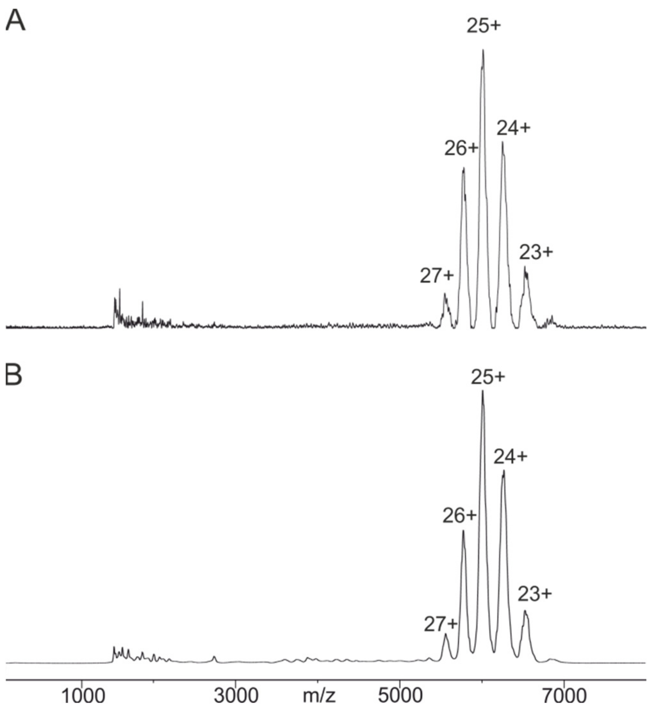

Figure S10. ITEM—SIX analysis, negative control. Nano-ESI mass spectra of tryptic Ovalbumin peptide mixture (solution 2´) plus IgG mixture prepared from rabbit serum (solution 0). **A:** ΔCV: 3 V. **B:** ΔCV: 20 V. Ion transmission below m/z 1450 is completely suppressed. Charge states are provided for the multiply charged ion signals of the IgG mixture. Protein concentration is 0.20 μg/μL. 2.5 μL were loaded. Solvent: 200 mM ammonium acetate, pH 6.7. Quadrupole vacuum: 3.9 × 10^{-5} mbar. Recording time per spectrum is 1 min. 40% baseline subtraction.
